# A New Unifying Definition of Resilience Adapted to the Developmental Psychopathology of Major Psychiatric Disorders: A Requisite for Progress

**DOI:** 10.1177/07067437251412627

**Published:** 2026-01-08

**Authors:** Yasmine Nadifi, Ileana Andrada Popa, Laurence Brochu, Gabrielle Girard, Michel Maziade

**Affiliations:** 1 33499 CERVO Brain Research Center, Quebec, Canada; 2 Department of Psychiatry and Neuroscience, 12369 Faculty of Medicine, Université Laval, Quebec, Canada

**Keywords:** resilience, protective factor, child and adolescent psychiatry, childhood adversity, sensitive period, high-risk youth, prevention, psychopathology, recalibration, buffering

Mounting evidence supports a developmental origin of schizophrenia, bipolar disorder and major depressive disorder.^1–3^ Understanding how these disorders emerge across development is therefore central to elucidate pathophysiology,^3,4^ and the most informative approach to date is to examine the trajectory of children and adolescents at familial high-risk. However, most longitudinal studies have concentrated on risk factors with limited attention to the protective mechanisms that may counterbalance the effect of risk factors. This questions the true predictive value of the longitudinal risk indicators previously found.^1,3–7^ Notably, two reviews published in this Journal 15 years ago emphasized that resilience is a dynamic, lifelong process and highlighted the lack of comparability among resilience studies.^8,9^ Since then, the field has made little progress for a variety of reasons.

In that respect, and from a clinical and developmental perspective, we identified four major reasons or limitations that hindered progress.^10^ The wide variability in the definitions and concepts of resilience – An understanding of resilience without an adversity lens – Resilience is neither only innate nor only learned – Strong dependence of protective mechanisms on sensitive periods. At the end of this article, we will propose a new unifying empirical framework for defining resilience which can enhance reproducibility across studies and ultimately inform clinical practice aimed at identifying and supporting vulnerable youth before illness onset.

## Variability in the Definitions of Resilience

Resilience has been studied since the 1970s. To date, no definition has emerged or been used consistently across studies.^9–11^ For some, resilience is difficult to operationalize and the definitions are either overly elaborate or too simple, often reduced to a positive adaptation to stressors.^12,13^ Historically, resilience has been conceptualized as the absence of pathology, though the field is moving away from such a dichotomous concept.^10,14^ Articles have highlighted the need to include risk in the context of resilience and have proposed the latter as a positive adaptation to significant adversity and a demanding environment.^15^ These ideas imply that resilience processes are context-dependent, shaped by the nature of adversity itself – an insight that should influence future research.

In parallel, several authors have described resilience as a personality trait, « an intrinsic and stable attribute ».^16^ In contrast to this static view, a growing body of work portrays resilience as a dynamic process.^9,11,17–19^ The latter notion implies a developmental perspective, recognizing that resilience emerges, fluctuates and transforms across different stages of life. As we will discuss later, timing is central to the definition of resilience. As Fritz et al. put it, resilience has measurable waxing and waning effects in response to risk factors rather than a static cross-sectional effect, which requires longitudinal designs to assess the process and detect the effects of resilience-enhancing factors.^18^ Moreover, defining resilience solely through internal personal strengths overlooks the well-documented interplay between internal attributes and environmental factors – such as social systems, family, school and community – in fostering resilience.^10,11,20^

In conclusion, resilience remains inconsistently conceptualized due to key limitations: an overly binary outcome-based framework, a narrow focus on individual traits that downplays environmental protection and the neglect of a developmental perspective.

## Understanding Resilience Through an Adversity Lens

Looking through an adversity lens implies the concept of stress.^9^ Stress is an intrinsic or extrinsic stimulus that triggers a biological response.^21^ Several authors have described a relationship between the amount, frequency and intensity of stressful events and the ability to develop resilience.^21^ In this light, stress is not merely a risk factor but also a driver of adaptation.^21^ This raises the central question of whether adversity is *necessary for developing* resilience or is only useful *for assessing* resilience.^17^

Three levels of stress exposure – *positive, tolerable, and toxic –* have previously been proposed.^22,23^ It must be acknowledged that the distinction between positive, tolerable and toxic stress is inherently subjective and context-dependent but can help illustrate how resilience must be interpreted under adverse or stressful circumstances. Accordingly, “positive stress” refers to a brief, moderate stressor, which allows the child to reach a state of balance following the activation of the stress response.^22^ An example of a normative stressor is a conflict within an interpersonal relationship. “Tolerable stress” involves a higher level of threat that is buffered by a protective factor that would help the child to physiologically recover. It is noteworthy that the same stressor may be experienced as tolerable by one child and toxic by another, depending on individual, relational and contextual variables. An example is the loss of a father, which might be considered a tolerable stress when a child benefits from a benevolent and supportive relationship with the mother and as a toxic stress for a child who does not have such support. The buffering theory here stipulates that intervening protective factors can mitigate the negative effects of exposure to a given risk.^24^ “Toxic stress” may then be more likely in the presence of frequent and intense adversity without a buffering factor.^22,23^ In this sense, depending on the timing, toxic stress can lead to structural and physiological changes in the brain that increase the vulnerability to behavioural and learning difficulties, and the subsequent development of psychiatric disorders.^23^

Compensatory mechanisms and recent research on the effect of childhood trauma on the brain are also instructive here.^25^ Teicher et al. found that adults exposed to maltreatment in childhood who did not later develop a psychiatric illness surprisingly displayed the same known brain neurobiological alterations caused by trauma as the exposed individuals who did.^26^ The explanation given is that individuals who remained healthy benefited from mechanisms that compensated for, rather than reversed, the neurobiological alterations. In other words, a compensatory process in some vulnerable individuals would alter brain connectivity in specific brain regions and then promote resilience.^26,27^

In conclusion, resilience has roots in the individual capacities and in the environment that shapes it. A proper understanding of resilience and the effect of protective factors then requires an appraisal of the intensity, type, frequency and timing of stress exposure.^27^

## Resilience is Neither Only Innate nor Only Learned

Contrary to the still widespread belief, a protective factor with a neurobiological basis does not exclude the possibility of modifiable properties.^11^ For example, cognitive skills or child temperament, although having definite hereditary components, can be positively modulated by the environment.^8,28,29^

One corollary is that, for achieving one's potential for resilience, an individual needs not only the corresponding biological characteristics but also those of the family and social environment to foster it. As a case in point, self-esteem, a well-documented protective factor, has apparent innate roots and can be enhanced or diminished by the influences of the family context or other environmental factors.^30^ Similarly, a positive longitudinal attachment to a caregiver in early life can buffer the effects of exposure to adversity.^9,24,31–33^ Maternal warmth mediates the relationship between low family socioeconomic status and poor health outcomes particularly caused by an inflammatory activity.^34,35^ Paternal emotional support may also contribute to resilience by providing support or teaching cognitive strategies in children of depressed mothers.^35,36^ More research is needed on the development of parenting skills, parental mentalization and a positive child-parent relationship for enhancing the full resilience potential of vulnerable children.

Remarkably, community-based protective factors have been overlooked as targets of interventions, such as school-based resilience-focused interventions targeting not only youth's internal resources but also schools that deal with inequalities.^29,37^ Community assets or resources, such as food, medical care, housing, income, schools, tutors, books, recreational facilities, neighbourhood safety and effective teachers appear to be critical in cultivating resilience.^9,38^ Social cohesion, a key community protective factor, i.e., the presence of shared values such as a common desire for safety and order, or a sense of good collective agreement, enhances the ability to develop resilience.^20^ In this regard, the community can promote trustworthy sources of support, particularly the presence of relationships with a significant adult outside the family, for children exposed to adversity.^8,20^ School-based resilience-focused interventions have ameliorating effects on outcomes such as depressive symptoms, internalizing and externalizing problems, and general psychological distress.^37,39^

In summary, even when having innate roots and being conceived as driven by individual factors, resilience is enhanced by specific and non-specific favourable familial and community environments.^18^ Resilience has to be addressed through a multisystemic approach. Clinicians need to promote interventions that enhance parenting skills and encourage healthy and significant relationships outside of the family. Resilience in at-risk youth can emerge at its best when familial and community resources are available.

## Protective Mechanisms Depend on Sensitive Periods

The timing of exposure to adverse events influences child outcomes.^40^ In contrast, the timing resilience growth throughout life has mostly been ignored. A proper definition of a sensitive time period incorporates a temporal interval during which environmental inputs have a better effect on brain plasticity.^7,40^ This definition challenges the common belief that the earlier the positive childhood experiences the greater the power in altering a child's developmental trajectories. *Three existing theories* are useful to remember when approaching sensitive periods of increased expression of resilience in the face of adversity: the *multiple hits*, the *recalibration* and the *buffering effect* models.

First, the “*multiple hits” theory* does not imply that the development of a major psychiatric disorder is necessarily due to the earliest risk events, but rather that the interaction of genetic factors and successive negative environmental factors over the course of adolescence increases the likelihood of a later transition to a major psychiatric disorder.^41–43^ For example, studies suggest that there are at least two major sensitive periods, the first “hit” being the impact of factors during the prenatal and perinatal periods, and the second “hit” consisting of risk agents coming into play in adolescence.^41,42^ Also, longitudinal data about grey matter abnormalities in patients with childhood-onset schizophrenia and their siblings suggests that protective factors that are active in late adolescence may elicit normalization of prefrontal and temporal grey matter abnormalities and thus prevent psychosis.^44,45^

Second, the “pubertal stress recalibration hypothesis” posits that, in case of exposure to adversity severe enough to mark the child's neurobiology, the subsequent effect of a protective factor during adolescence may counteract the adverse effect by wiping out some or all of the negative neurobiological patterns.^7,31,46^ Other telling examples are the longitudinal studies of children who were institutionalized in infancy and later benefited from a stable and supportive family environment during adolescence.^31,32^ The latter positive experience would restore the functioning of the hypothalamic-pituitary-adrenal axis by recalibrating cortisol to levels like those of control children who were not institutionalized.^31,32^ Protective factors recalibrating previous adverse effects during sensitive developmental periods may guide a future leading to preventive actions.^46^

Third, the *buffering effect* theory, described previously, contrasts with the recalibration hypothesis in the nature of timing since it requires the protective factor to be contemporaneous to the exposure to adversity for reducing the impact of the risk factor.^22–24^ The theory stipulates that a buffering effect against stress varies in effectiveness depending on specific developmental stages,^24,47^ such as the transition to adolescence^14,33,48–50^ in which a decrease in the effectiveness of parental support would be partly replaced by peer support.^47,51^

Sex differences in sensitive periods also exist for specific adverse factors such as the type and timing of trauma, particularly when adolescents enter heightened brain plasticity.^52^ Given that males and females show different predispositions to psychopathology, biological sex matters with respect to differential efficacy of protective factors modifying the childhood risk trajectory.^53,54^ A consequential bias to be corrected is the lower number of females included in risk research protocols, a central problem to address for future translation to the clinic.^37,53,55^

In sum, the search for the second or third adverse hit in high-risk children exposed to a first hit should influence the methods of future research aiming to decrease the likelihood of a later transition to a major disorder.^7,33^ Pubertal recalibration heightens the relevance of periods of enhanced neuroplasticity. Buffering mechanisms would vary according to developmental periods. Time frames of enhanced potential for resilience are likely to provide time-specific interventions which will inform the clinical surveillance of children and adolescents in primary care.

## A Unifying Multidimensional Definition of Resilience

It is vital for progress to standardize nomenclature to achieve a consensual language for research and clinical practice. The four obstacles we described and the existing evidence can lead to the following new comprehensive definition of resilience in childhood and adolescence: i) a developmentally malleable and dynamic process, ii) having environmental and innate roots, some of which may be sex- or risk-specific factors, iii) emerging from childhood in reaction to adversity, iv) provoking the expression of multisystemic adaptive mechanisms that allow the young individual to achieve better-than-expected functional outcomes, and v) the emergence of which depends on specific developmental periods of enhanced plasticity. This definition implies windows of opportunity for tailoring preventive and corrective interventions focused on resilience with greatest potential for benefits.
